# Using data from multiple studies to develop a child growth correlation matrix

**DOI:** 10.1002/sim.7696

**Published:** 2018-04-26

**Authors:** Craig Anderson, Luo Xiao, William Checkley

**Affiliations:** ^1^ School of Mathematical and Physical Sciences University of Technology Sydney Ultimo NSW 2007 Australia; ^2^ ARC Centre of Excellence for Mathematical and Statistical Frontiers Parkville Victoria Australia; ^3^ Department of Statistics North Carolina State University Raleigh NC 27695 USA; ^4^ Division of Pulmonary and Critical Care, Department of Medicine Johns Hopkins University Baltimore MD 21218 USA; ^5^ Program in Global Disease Epidemiology and Control, Department of International Health Bloomberg School of Public Health Johns Hopkins University Baltimore MD 21218 USA

**Keywords:** child health, correlation, growth, SDS

## Abstract

In many countries, the monitoring of child growth does not occur in a regular manner, and instead, we may have to rely on sporadic observations that are subject to substantial measurement error. In these countries, it can be difficult to identify patterns of poor growth, and faltering children may miss out on essential health interventions. The contribution of this paper is to provide a framework for pooling together multiple datasets, thus allowing us to overcome the issue of sparse data and provide improved estimates of growth. We use data from multiple longitudinal growth studies to construct a common correlation matrix that can be used in estimation and prediction of child growth. We propose a novel 2‐stage approach: In stage 1, we construct a raw matrix via a set of univariate meta‐analyses, and in stage 2, we smooth this raw matrix to obtain a more realistic correlation matrix. The methodology is illustrated using data from 16 child growth studies from the Bill and Melinda Gates Foundation's Healthy Birth Growth and Development knowledge integration project and identifies strong correlation for both height and weight between the ages of 4 and 12 years. We use a case study to provide an example of how this matrix can be used to help compute growth measures.

## 1 BACKGROUND

The study of physical growth in children is a challenging and complex topic that must consider a variety of genetic, physiological, and socio‐economic factors. There is a vast literature on child growth, and a thorough overview of the topic is provided in two books by J.M. Tanner.^1^, ^2^ This paper will focus on the estimation and prediction of growth based on data from longitudinal growth studies. There is a great deal of interest in being able to understand the factors that drive growth faltering in young children, in order to develop better preventative measures.^3^, ^4^, ^5^

To adequately monitor the health of young children, it is imperative that we are able to accurately model their growth across their formative years. In many countries, children are measured regularly to track their progress, but there are many places where such monitoring does not occur in a consistent or regular manner.^6^ In these countries, a child's height and weight may only be measured sporadically, and these measurements may be subject to a great deal of measurement error. It can thus be very challenging to estimate the growth pattern of children in these areas and to identify children whose growth may be faltering and who may require health interventions. By pooling together multiple datasets and drawing strength across studies, it may be possible to overcome data sparsity issues and provide substantially better estimates than would be possible from a single study.

To estimate a child's growth where measurements are sparse, it is important to have some measurement of how much correlation exists between a child's growth measures across different ages.^7^ For example, we would be keen to know whether a child's height at age 100 days is likely to be a good predictor for that same child's height at 300 days. If we know the correlation between these time points in a child's life, then we are able to make inference about what height a child might be after 300 days given that we know their height at 100 days. Such predictions can also help to quickly identify whether a child's growth is faltering—if their actual observed height at 300 days is substantially less than what was predicted, then this could indicate poor growth over that period,^2^, ^8^ which may be a result of an underlying health issue.^9^, ^10^, ^11^ This approach is common in child health monitoring; the World Health Organisation (WHO) have developed a set of widely used growth reference charts^12^ that show a healthy range of heights and weights for children at each age point, thus allowing parents and doctors to quickly identify when a child is developing more slowly than would be expected.

The existing WHO growth curves were built using a mixture of longitudinal data measured from birth to 24 months and cross‐sectional data from children aged 18 to 71 months. Therefore, these curves may not reflect longitudinal trends for children aged 24 months and older as accurately as would be the case with longitudinally observed data. The approach outlined in this paper incorporates both subject‐specific and marginal correlation data to provide an estimate of a child's growth curve. By using longitudinal data from low‐ and middle‐income countries, we aim to produce a more realistic, empirical representation of growth trajectories for these children. A well‐developed correlation matrix using growth data from resource‐poor settings would be a useful resource to help medical experts provide accurate growth estimation for children in such countries. Such a correlation matrix also helps facilitate the computation of conditional standard deviation scores (cSDS), which measure the relative change in height from one time point to the next (eg, a change in height between 100 and 300 days in the example above). In this paper, we propose a novel 2‐stage approach, which uses data from multiple studies to construct a common correlation matrix that can be used in estimation and prediction of child growth. The first stage involves the construction of a raw and often incomplete matrix via a set of elementwise univariate meta‐analyses. In stage 2, we smooth this raw matrix in order to obtain a valid and complete correlation matrix.

## 2 DATA

As part of the Bill and Melinda Gates Foundation's Healthy Birth Growth and Development knowledge integration project, we have access to 16 studies, which include sufficient longitudinal child growth data. Data from the following studies were used in this paper: Evaluation and Control of Neglected Mucosal Enteric Infections in Childhood (*cntt*, European Commission^13^); lower respiratory tract infection (LRTI), respiratory syncytial virus (RSV), and Influenza Cohort Study (*grip*, Iqbal et al^14^); PROVIDE Study PR‐10060, funded by NIH grant R01 AI043596 (*prvd*, Naylor et al^15^); Infant Growth in Peru (*phua*, Lopez de Romaña et al^16^); Respiratory Pathogens Birth Cohort (*rspk*, Iqbal et al^14^); Peru Zn Fortification (*pzn*, Brown et al^17^); Longitudinal study of bovine serum concentrate (BSC) in Guatemala (*gbsc*, Begin et al^18^); Medical Research Council (MRC) Keneba (*knba*, Hennig et al^19^); Study of Biomarkers for Environmental Enteropathy (*ee*, Iqbal et al^14^); Deuterium dilution study in Mali (*mmam*, Ackatia‐Armah et al^20^); Child Malnutrition and Infection Network (*cmin*, MAL‐ED Investigators^21^); Zn Trial in Burkina Faso (*bfzn*, Hess et al^22^); CMC Vellore Birth Cohort 2002 (*cmc*, Rehman et al^23^); NIH Birth Cohort Study, funded by NIH grant R01 AI043596 (*nbrt*, Mondal et al^24^); Longitudinal Growth Study in Bangladesh (*bngd*, Brown et al^25^); NIH Preschool Cohort Study, funded by NIH grant R01 AI043596 (*npre*, Haque et al^26^).

Table 1 provides a summary of the data within these studies. Within each study, a group of children have their height and weight measured at a number of different ages. The measurement ages and the number of measurements are not consistent from child to child, even within a study. For example, child A may be measured at ages 100, 200, and 300 days, while child B may be measured at ages 150 and 250 days. Each study covers a slightly different age range of children; for example, *bfzn* contains children aged between 168 and 927 days, while *grip* contains children aged between 1 and 521 days. It is also likely that the amount of measurement error will vary from one study to the next, since the studies were conducted independently in a variety of locations, using different study methodologies and measurement techniques. Our model includes a parameter that accounts for this difference in data quality between studies.

**Table 1:** Summary of relevant studies within the HBGDki project

Dataset	# Children	# Obs	Obs Per Child			Child Age (in Days)		
			Min	Median	Max	Min	Median	Max
cntt	197	4405	10	21	41	1	116	702
grip	203	1427	1	7	17	1	136	521
prvd	700	9741	1	16	16	1	175	756
phua	153	1839	1	13	16	1	185	679
rspk	278	3177	1	13	33	1	211	525
pzn	302	1140	2	4	4	153	265	457
gbsc	315	2548	1	10	13	119	269	493
knba	2954	41587	1	13	69	1	309	900
ee	380	8436	2	23	26	1	343	1175
mmam	289	577	1	2	2	186	423	1090
cmin	3125	35506	1	9	37	1	446	1846
bfzn	7637	18983	1	2	4	168	541	927
cmc	373	12478	23	34	37	1	558	1111
nbrt	629	11828	1	21	43	1	644	2199
bngd	197	2352	1	14	15	95	804	1903
npre	529	8656	1	16	30	731	3257	6696

Abbreviation: HBGDki, Healthy Birth Growth and Development knowledge integration.

## 3 METHODS

The heights and weights in every study were standardised using the WHO's Z‐scores.^12^ A Z‐score reflects a child's development relative to the global “healthy” average. In this paper, we will work with both height‐for‐age Z‐scores (HAZ) and weight‐for‐age Z‐scores (WAZ). Some applications also use weight‐for‐height Z‐scores, but those are not analysed in this study. One issue with standardisation across multiple geographical regions is that there may be differing secular trends in growth around the world, which may have an impact on estimation and prediction.^27^ However, standardisation of the data facilitates direct comparison between children of different ages, heights, and weights, which is important when comparing multiple studies.

The rate of a child's growth, often referred to as growth velocity, is commonly assessed via centile‐crossing approaches.^28^ Such approaches characterise a child's growth velocity based on the change in their relative height or weight between 2 time points. Across the study, the expected mean change in Z‐score will be 0 as long as an appropriate growth reference is used. If a child has a Z‐score of 0.2 at age 100 days, but a Z‐score of 0.5 at age 200 days, then that implies that they have grown quickly relative to their peers over that period. However, it is well known that such measures can be affected by regression to the mean since smaller children are more likely to grow relatively quickly and larger children are more likely to grow relatively slowly.^29^ The cSDS accounts for this by adjusting for the correlation between the 2 time points of interest.

Let *Z*_1_ and *Z*_2_ be the Z‐scores measured at ages *t*_1_ and *t*_2_, respectively, and let *r*_12_ be the correlation between these 2 time points. Then, the cSDS between these time points, denoted *Z*_⟨2|1⟩_, is given by

(1) $$Z_{\left\langle 2|1\right\rangle }=\frac{Z_{2}-r_{12}Z_{1}}{\sqrt{1-r_{12}^{2}}}.$$

Note that this velocity does not directly depend on the time points *t*_1_ and *t*_2_, but instead on the correlation between them, *r*_12_. It is therefore crucial that we are able to accurately estimate this correlation term for all pairs of possible time points to facilitate the calculation of growth velocities. To compute such correlations, it is first necessary to discretise continuous time into a series of age groups. The nature of this discretisation will be dependent on both the context of the study and the computational time available. In practice, it should be sufficient to construct the matrix based on age groups within which the correlation is likely to be constant and stable, based on existing knowledge of growth patterns.

Developing an overall correlation matrix based on these studies is difficult. Ideally, one might wish to combine all of the data into 1 large dataset and then construct the underlying correlation structure in a single stage using some form of multilevel model. However, the sheer volume of data within these studies makes this approach computationally challenging; we have over 100 000 observations taken from over 15 000 children. Instead, we consider a 2‐stage approach where we fit a separate correlation matrix for each study and then combine these matrices into one larger matrix. One important advantage of this 2‐stage approach is its ability to easily handle new datasets. If we wish to integrate the results from a new study into our matrix, then we simply have to construct the correlation matrix for that study and then update our final matrix. Under a 1‐stage model, we would have to repeat the entire analysis each time a new study was added.

It is fairly straightforward to compute study‐specific correlation matrices in studies that have a regular and structured observation schedule.^7^ However, it is more challenging to do so in studies such as ours, which have a sparse and/or irregular data structure. One possibility is to assume that the correlation matrix takes a parametric form with a few unspecified parameters, eg, Argyle et al.^30^ However, model misspecification can be a potential issue. In recent years, functional data analysis has been widely used for analysing longitudinal data.^31^ The advantage of functional data analysis is that it only requires that the correlation matrix is smooth across age, which seems reasonable for child growth curves, and does not impose any parametric form on the correlation matrix. Under the functional data model, a smooth correlation matrix can be obtained by conducting a bivariate smoothing of empirical covariances; see Appendix A for details. To obtain smooth correlation matrices, we used the fast covariance estimation method proposed in Xiao et al,^32^ which was specifically designed for longitudinal data. The fast covariance estimation method was implemented in the *face* R package.^33^ This method allowed us to estimate smooth covariance matrices for both height and weight Z‐scores in each of the 16 studies.

**Figure 1:**
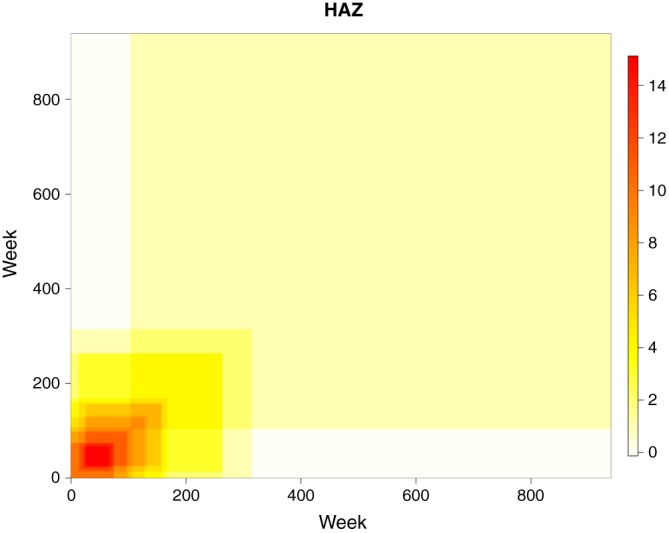
A heat map showing the extent of the correlation matrices for individual studies. The colour corresponds to the number of matrices that cover that particular age range. Darker red means more studies are available, while paler yellow means fewer studies are available. HAZ, height‐for‐age Z‐scores [Colour figure can be viewed at wileyonlinelibrary.com]

We then have 16 HAZ correlation matrices and 16 WAZ correlation matrices that we would like to combine to give a single HAZ and a single WAZ matrix, which can describe all children across all 16 studies. However, each individual matrix only describes the correlations for children within the age range of that particular study. The differences between the 16 studies mean that each of these 16 matrices covers a different age range. Figure 1 displays a heat map of all the study‐specific correlation matrices across the 0‐ to 6570‐day age range (this corresponds to 0 to 18 years). The colour corresponds to the number of studies that cover that particular age range, with darker red corresponding to more studies being available and lighter yellow meaning that fewer studies are available. We can see that we have lots of data available for the younger age groups, but less data are available for the older ages. Only 1 study (*npre*) covers the area in the top right corner of the matrix (roughly 4000‐6570 days), while there is no information available for the areas in the top left and bottom right of the plot (this corresponds to the correlation between very young children and older teenagers).

There are 2 basic approaches to meta‐analysing correlation matrices. The first is the univariate approach, where the meta‐analysis of the matrix is conducted on an elementwise basis; for example, a 10×10 matrix would require 100 separate meta‐analyses.^34^ The second is a multivariate approach, which attempts to combine the matrices as a whole.^35^ The multivariate approach has a number of advantages, most notably in terms of being able to maintain the smoothness of the correlation matrix,^36^ but the methodology has been developed to handle a set of homogeneous matrices, and it is not clear how to adapt this methodology to deal with our case where each matrix covers a slightly different range. With that in mind, we adopt a univariate approach for our meta‐analysis.

## 4 STATISTICAL METHODOLOGY

Let *A*=*a*_1_,…,*a*_*r*_ be the range of ages across which we wish to estimate correlations, with *A* typically defined by the age range of the children across all studies. We thus wish to estimate the combined correlation matrix *H*, where *H* is an *r*×*r* matrix with entry *h*_*ij*_ corresponding to the correlation between children aged *a*_*i*_ and *a*_*j*_.

Suppose that we have *K* studies, indexed by *k*=1,…,*K*. Let *n*_*k*_ be the number of children who were observed in study *k*, and let $B_{k}=b_{k1},\text{⋯},b_{ks_{k}}$ be the range of ages across which we have estimated correlations for study *k*, where *s*_*k*_ is the number of discrete timepoints within the scope of the study. Assume that we have the set of matrices *C*={*C*_1_,…,*C*_*K*_}, where *C*_*k*_ is the *s*_*k*_×*s*_*k*_ correlation matrix from study *k*, with *c*_*ijk*_ corresponding to the correlation between children aged *b*_*ki*_ and *b*_*kj*_ for study *k*. Since *B*_*k*_ and *s*_*k*_ may vary from study to study, the corresponding correlation matrices also differ in terms of size and scope, and it is thus important that we retain consistent indexing when estimating *H*. Thus, we construct a set of matrices $\tilde{C}=\{{\tilde{C}}_{1},\text{⋯},{\tilde{C}}_{K}\}$, where each ${\tilde{C}}_{k}$ is an *r*×*r* matrix with entries defined as follows:

$${\tilde{c}}_{ijk}=\left\{\begin{array}{cc} c_{ijk} & \;\text{if}\;i,j\in B_{k}\\ . & \;\text{otherwise,} \end{array}\right.$$

We now have a set of *K* consistently indexed study‐specific correlation matrices, and we wish to combine these into a single matrix *H*. This is achieved via a 2‐stage process: First, we conduct univariate meta‐analysis to construct a rough, incomplete correlation matrix, and then we apply a bivariate smoother to produce a more realistic estimate of *H*.

### 4.1 Univariate meta‐analysis

The univariate meta‐analysis approach allows us to use a set of elementwise calculations to construct a rough estimate $\tilde{H}$ for the combined correlation matrix, based on a set of elementwise calculations. Here, each element ${\tilde{h}}_{ij}$ is computed separately based on the set of values ${\tilde{c}}_{ij1},\text{⋯},{\tilde{c}}_{ijK}$. Our univariate meta‐analysis is based on the random effects approach outlined by Hedges and Olkin.^34^ The random effects approach has an underlying assumption that the set of observed correlation matrices $\tilde{C}$ form a sample from a larger population of correlation matrices, which is an entirely reasonable assumption in our context since we only observe a small sample of all the children in the world. This model makes an assumption that each of the correlations come from a normal distribution, and therefore, a Fisher transform is applied to each correlation to ensure normality.^37^ The set of Fisher‐transformed matrices is denoted by $\tilde{F}$.

Since our study‐specific correlation matrices $\tilde{C}$, and thus our Fisher‐transformed matrices $\tilde{F}$ were themselves estimated via a model, we assume that each observed value ${\tilde{f}}_{ijk}$ is an unbiased estimate of the true study‐specific correlation *Θ*_*ijk*_. We assume the model

$${\tilde{f}}_{ijk}=\Theta_{ijk}+\varepsilon_{ijk},$$

where $\varepsilon_{ijk}∼N(0,\sigma_{ijk}^{2})$. By ensuring that each correlation observation has its own error term, we can account for the differences in data quality between studies. We further assume that each of these study‐specific correlations *Θ*_*ijk*_ comes from a normal distribution with mean *m*_*ij*_ and a variance *τ* ^2^. Here, *τ* ^2^ represents the level of heterogeneity between the studies. This leads to the random effects model

$${\tilde{f}}_{ijk}=m_{ij}+\varphi_{ijk}+e_{ijk},$$

where *φ*_*ijk*_∼N(0,*τ* ^2^).

We obtain our estimate ${\tilde{m}}_{ijk}$ via a weighted average of the true study‐specific terms *Θ*_*ijk*_ based on the random effects model. Here, we use inverse‐variance weights,^34^ given by

$$w_{ijk}=\frac{1}{\sigma_{ijk}^{2}+\tau^{2}}.$$

This weighting structure ensures that as the uncertainty associated with a correlation observation increases, the weight given to that observation decreases. Our weighted average takes the form

$${\tilde{m}}_{ij}=\frac{\sum_{k=1}^{K}w_{ijk}\Theta_{ijk}}{\sum_{k=1}^{K}w_{ijk}}.$$

Applying this approach to each possible pair (*i*,*j*), we obtain a matrix, *M*. An inverse Fisher transformation is then applied to *M* to obtain the final rough correlation matrix *H*.

Note that in practice, we do not always have estimated correlations ${\tilde{c}}_{ijk}$ for all *k*. In the case where the correlation between ages *i* and *j* was not estimated in a specific study, this study is not included in the estimation of ${\tilde{h}}_{ij}$. In the case where the correlation between ages *i* and *j* was not estimated in any studies, we do not obtain an estimate for ${\tilde{h}}_{ij}$ and must compute this using the smoothing technique outlined in the next section.

### 4.2 Smoothing

The matrix obtained in stage 1 provides a rough estimate of the correlation structure but has 2 major flaws. The matrix may be incomplete due to a lack of data covering a pair of ages (see Figure 1 for an illustration), which means it cannot be used as a correlation matrix. Additionally, the matrix is likely to be subject to large discontinuities in the correlation surface due to the univariate nature of the estimation procedure. These 2 issues can be addressed by smoothing the matrix to provide a more realistic correlation surface.

To obtain smooth estimates of correlation functions on the off‐diagonals and also to fill in unobserved correlations, we conduct a bivariate smoothing on the existing correlations. Let $({\tilde{h}}_{ij},a_{i},a_{j},\delta_{ij}),1\leq i\leq r,1\leq j\leq r$ be the estimated correlations from stage 1, where ${\tilde{h}}_{ij}$ is the estimated correlation at ages *a*_*i*_ and *a*_*j*_ and *δ*_*ij*_ is 1 if ${\tilde{h}}_{ij}$ exists and 0 otherwise.

We first conduct a Fisher transformation of these correlations, $g_{ij}=\frac{1}{2}\ln\left(\frac{1-{\tilde{h}}_{ij}}{1+{\tilde{h}}_{ij}}\right)$. Then, we conduct a bivariate smoothing of *g*_*ij*_ under the working model *g*_*ij*_=*g*(*a*_*i*_,*a*_*j*_)+*ε*_*ij*_, where *g*(*a*_*i*_,*a*_*j*_) is a bivariate smooth function and *ε*_*ij*_ is independent Gaussian random variables. We use the spline smoother outlined in Marx and Eilers,^38^ with a constraint added to ensure the symmetry of our eventual correlation function. We obtain an estimated function $Ĝ(a_{i},a_{j})$ and apply the inverse Fisher transform to $Ĝ(a_{i},a_{j})$ to obtain our correlation estimate ${\hat{\rho }}_{ij}$. This process is described in more detail in Appendix B.

To ensure the integrity of the correlation matrix, we remove the diagonal elements (which are 1s) prior to the smoothing and then normalise the smoothed correlations to ensure that the final matrix has 1s on the diagonal. Specifically, let $\hat{\Sigma }=({\hat{\rho }}_{ij})$ be the estimated correlation matrix after smoothing; then, the final matrix is $\tilde{\Sigma }=({\tilde{\rho }}_{ij})$, where ${\tilde{\rho }}_{ij}={\hat{\rho }}_{ij}/\sqrt{{\hat{\rho }}_{ii}{\hat{\rho }}_{jj}}$.

## 5 APPLICATION

We applied the methodology outlined in Section 4 to the set of 16 studies outlined in Section 3. The aim was to construct a large combined correlation matrix that covers the entire range of the set of studies (0 to 6570 days). Doing this on a day‐by‐day basis would have required a 6571×6571 matrix, which is extremely large and would require a great deal of computational time. We thus simplified matters by computing the correlations on a week‐by‐week basis, which corresponds to a more manageable 940×940 matrix. By doing so, we are making an assumption that correlation is stable and constant within a week, which seems reasonable. The choice of weekly age groups was made to ensure a sufficient level of detail for very young children, where rapid growth changes can occur. For older children, weekly age groupings are less necessary, but we have retained them to ensure a consistent structure.

**Figure 2:**
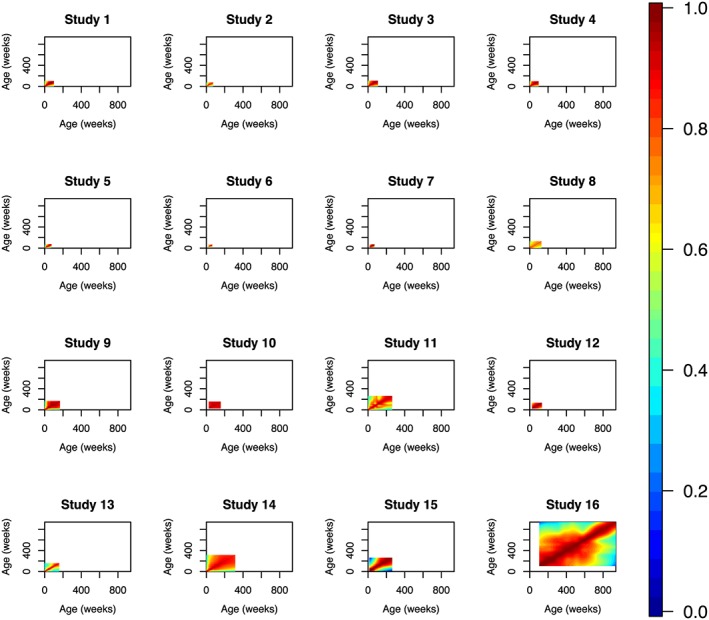
Week‐by‐week height‐for‐age Z‐scores correlation matrices for each of the 16 studies [Colour figure can be viewed at wileyonlinelibrary.com]

**Figure 3:**
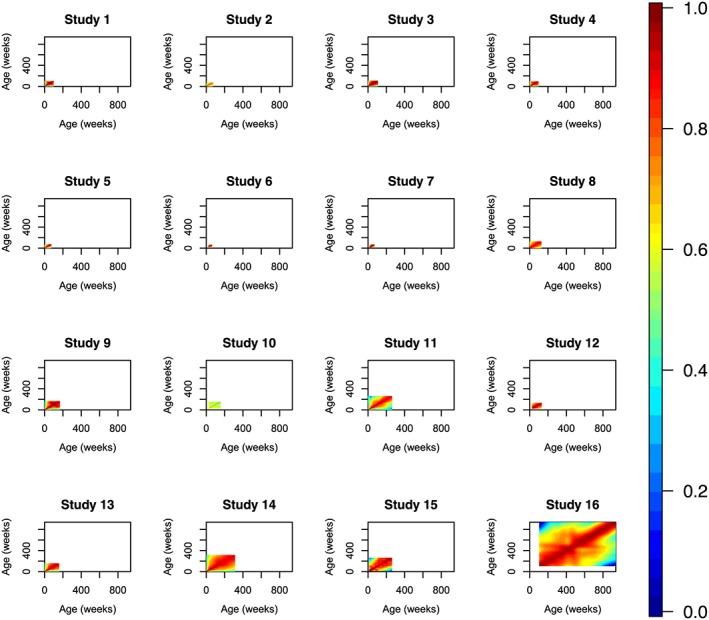
Week‐by‐week weight‐for‐age Z‐scores correlation matrices for each of the 16 studies [Colour figure can be viewed at wileyonlinelibrary.com]

The univariate meta‐analysis step outlined in section 4.1 was conducted for HAZ and WAZ in turn. The first step of this process was to construct separate HAZ and WAZ correlation matrices for each of our 16 studies, and these are illustrated in Figures 2 and 3. These matrices were then combined using the using the *metacor* function, which is part of the *meta* R package.^39^ We computed each entry of our 940×940 weekly correlation matrix $\tilde{H}$ in turn. In keeping with the methodology outlined in section 4.1, each entry of $\tilde{H}$ only took into account the studies that provided a correlation estimate for that age range. That means that some of the correlation parameters are estimated based on as many as 15 datasets, while others are estimated based on just 1 dataset, as illustrated in Figure 1. This also means that a small number of entries could not be estimated at this stage due to a lack of data in the initial studies.

**Figure 4:**
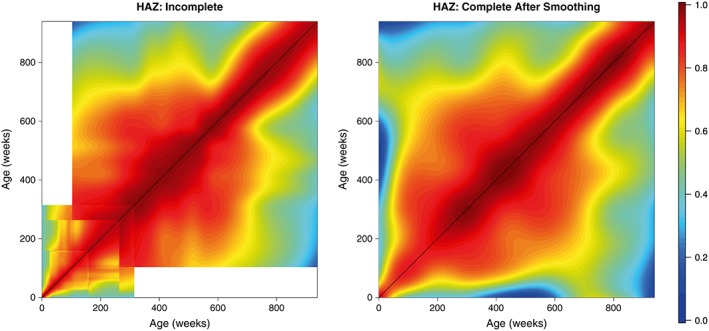
Week‐by‐week height‐for‐age Z‐scores (HAZ) correlation matrix obtained via meta‐analysis of 16 studies. The left panel displays the unsmoothed estimates obtained from the univariate meta‐analysis, while the right panel displays the final smoothed matrix. Dark red corresponds to high correlation, while blue corresponds to lower correlation [Colour figure can be viewed at wileyonlinelibrary.com]

**Figure 5:**
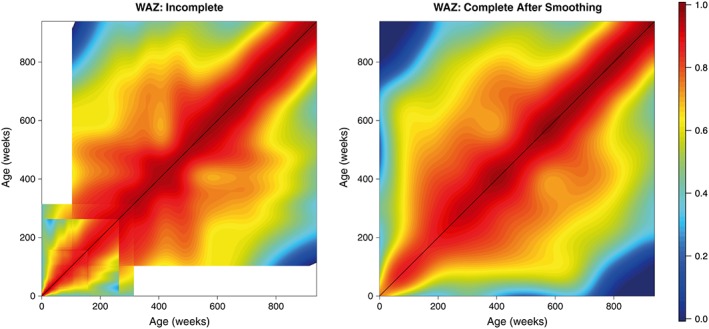
Week‐by‐week weight‐for‐age Z‐scores (WAZ) correlation matrix obtained via meta‐analysis of 16 Gates studies. The left panel displays the unsmoothed estimates obtained from the univariate meta‐analysis, while the right panel displays the final smoothed matrix. Dark red corresponds to high correlation, while blue corresponds to lower correlation [Colour figure can be viewed at wileyonlinelibrary.com]

The raw HAZ and WAZ correlation estimates obtained via this simple meta‐analysis are displayed in the left panels of Figures 4 and 5, respectively. Dark red corresponds to high correlation, while blue corresponds to lower correlation. Unsurprisingly, it appears that the correlations are higher for low age differences and get lower as the difference between the time points increases. We can also note that the matrices produced are far from smooth and thus may not provide an entirely realistic representation of the true correlation structure. The white regions correspond to the ages between which we were unable to compute correlations.

**Figure 6:**
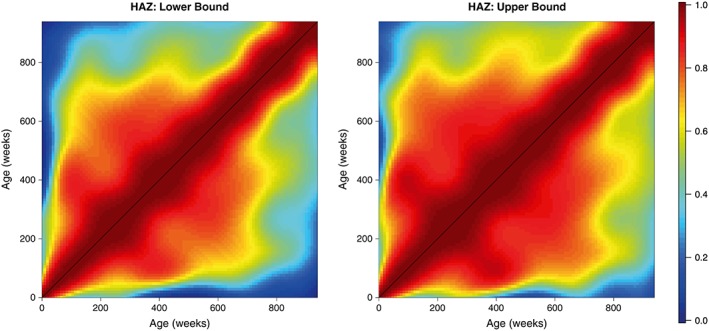
The lower and upper bounds of the height‐for‐age Z‐scores (HAZ) correlation matrix, calculated by smoothing the incomplete lower and upper confidence surfaces [Colour figure can be viewed at wileyonlinelibrary.com]

**Figure 7:**
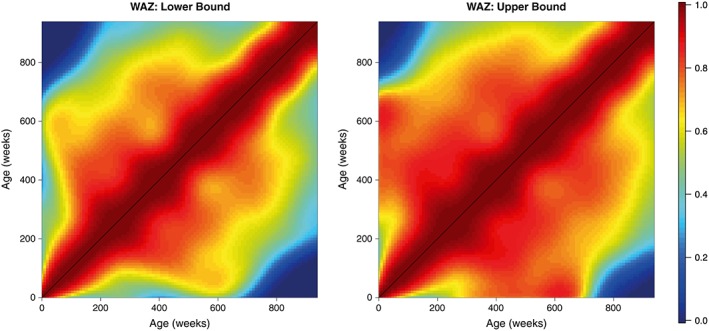
The lower and upper bounds of the weight‐for‐age Z‐scores (WAZ) correlation matrix, calculated by smoothing the incomplete lower and upper confidence surfaces [Colour figure can be viewed at wileyonlinelibrary.com]

**Figure 8:**
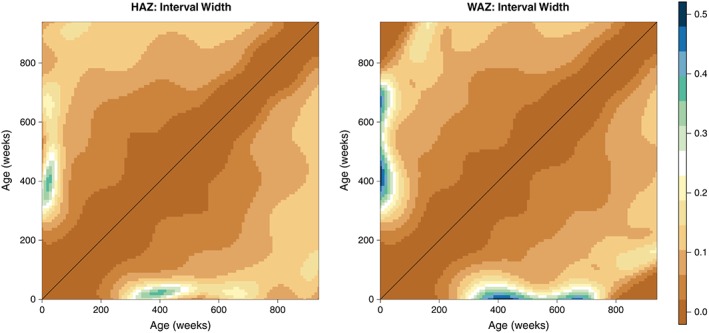
The uncertainty surface for our estimated height‐for‐age Z‐scores (HAZ) and weight‐for‐age Z‐scores (WAZ) matrices, displayed as the difference between lower bounds and upper bounds [Colour figure can be viewed at wileyonlinelibrary.com]

These matrices were then smoothed as outlined in section 4.2 to provide more realistic estimates of the correlation surface. These smoothed matrices are displayed in the right panels of Figures 4 and 5. In both cases, we obtain matrices that possess the necessary characteristics of a correlation matrix—they are complete, smooth, and symmetric; all values lie within the range 0 to 1; and all diagonal entires are equal to 1. We also obtain lower and upper confidence surfaces for both HAZ and WAZ by smoothing the incomplete lower and upper confidence surfaces obtained from our univariate meta‐analyses; these are displayed in Figures 6 and 7. Additionally, Figure 8 displays the uncertainty surface for each of our estimated correlation matrices, obtained by subtracting the lower bounds from the upper bounds.

We identify strong correlations over small age gaps, but these correlations reduce for larger age gaps. There appears to be very little correlation between one's height (or weight) as a baby and one's height (or weight) as an adult, which is not hugely surprising, but is still an interesting result. Reasonably strong correlations exist between the ages of 200 and 600 weeks (roughly 4 and 12 years), which suggests that children remain fairly stable in terms of height and weight prior to puberty. This is in line with existing results that note that after a child's initial early development, their growth remains roughly constant until puberty.^40^ These results suggest that growth in the early part of a child's life (up to 4 years) is crucial in a child's future development, since height and weight at age 4 appear to be excellent predictors for height and weight at age 12.

## 6 CASE STUDY—COMPUTING GROWTH VELOCITIES

In this section, we provide a case study that shows how our correlation matrix can be used to compute growth velocities. Recall from Equation 1 that the computation of the cSDS, *Z*_⟨2|1⟩_, is dependent on *r*_12_, a measure of the correlation between time points *t*_1_ and *t*_2_. Using the combined correlation matrix computed in Section 5, we can extract the estimated correlation *r*_12_ for any pair of time points *t*_1_, *t*_2_ in the range 0 to 6570 days. This will be illustrated using the data from *cntt*.

The study outlined in *cntt* was conducted in 2 peri‐urban shanty towns with high population density, just outside Lima, Peru. These peri‐urban communities are composed of 50 000 residents, the majority of whom are immigrants from rural areas. In the last 2 decades, this area has undergone many economic and social developments. The study contains 197 children with anthropometric measurements taken from birth. The median number of observations per child was 23, with a total of 4405 data points obtained.

**Figure 9:**
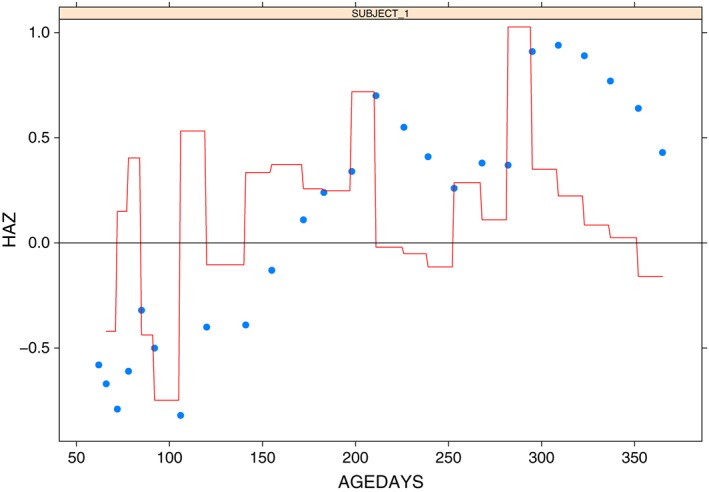
Visualisation of Z‐scores and conditional standard deviation score (cSDS) for a single child from cntt. The blue points represent observed Z‐scores, and the red line displays the cSDS computed between each pair of consecutive points. HAZ, height‐for‐age Z‐scores [Colour figure can be viewed at wileyonlinelibrary.com]

We will focus on 1 randomly selected child from this study and will show how the correlation matrix can be used to compute growth velocities. The selected child was observed 25 times during the study, and their Z‐scores are plotted as blue dots in Figure 9. Let *Z*_1_,…*Z*_25_ be the set of HAZ scores obtained for this child at timepoints *t*_1_,…*t*_25_. Suppose we wish to measure the growth velocity for this child between their first and last measurements. Here, *Z*_1_=−0.58, where *t*_1_=62 days, and *Z*_25_=0.43, with *t*_25_=365 days. To compute the cSDS, *Z*_⟨25|1⟩_, we also need *r*_1,25_, which is the correlation between 62 and 365 days. This correlation can be obtained from our combined correlation matrix as 0.66. Using these values, we can compute the cSDS as follows:

$$Z_{\left\langle 25|1\right\rangle }=\frac{0.43-0.66\times (-0.58)}{\sqrt{1-0.66^{2}}}=1.08.$$

This implies an overall improvement in the velocity score of around 1 standard deviation over the period of observation. Recall that the cSDS uses the correlation to account for regression to the mean, and therefore, this computed score should be independent of the child's initial height. It may also be of interest to calculate what we refer to as dynamic cSDS by computing the cSDS between each pair of consecutive observations, thus identifying changes in the child's rate of growth over the observation period. Figure 9 provides a display of this dynamic cSDS; the blue points represent the observed Z‐scores, and the red line corresponds to the dynamic cSDS. We can see that there is a general trend of growth over time, but we also note that there are some periods where the child's Z‐score decreases. By developing the growth matrix in Section 5, we have allowed users the flexibility to measure growth in both the short and long terms, thus allowing them to look at overall trends and also identify short periods of poor growth, which may merit further investigation.

To further illustrate the importance of our matrix in computing these scores, we randomly selected another 10 children from the study and computed the cSDS between their first and last observations. These results are displayed in Table 2. We note here that the inclusion of the correlation term to account for regression to the mean has a big impact on the scores. For example, child A has a very slight increase in Z‐score between ages 1 and 2 and yet ends up with a cSDS of −0.60. This is because one would expect a much more substantial improvement in child A as a result of regression to the mean, and a failure to improve may be an indication of ongoing poor growth. Compare this to child B, who has a very similar cSDS value despite a Z‐score decrease of 0.99. Child B's height is well above average to begin with, and therefore, a relative decrease is of much less concern than it would be for a child who had started off low and dropped further.

**Table 2:** Table of cSDS values for 10 randomly selected children

Child	Age 1, *t*_1_	Age 2, *t*_2_	*Z*_1_	*Z*_2_	Correlation, *r*_12_	**cSDS**, *Z*_⟨2|1⟩_
A	41	428	−1.23	−1.21	0.59	−**0.60**
B	15	365	1.18	0.19	0.60	−**0.66**
C	35	308	−2.37	−0.45	0.67	** 1.56**
D	36	397	−3.56	−1.18	0.60	** 1.21**
E	86	702	−2.58	−0.03	0.52	** 1.54**
F	44	265	−0.75	−0.53	0.72	** 0.01**
G	57	183	−40.45	−1.14	0.80	−**1.30**
H	22	182	−1.16	−0.65	0.78	** 0.41**
J	29	182	−2.11	−0.79	0.79	** 1.41**
K	29	308	−0.58	−0.71	0.67	−**0.43**

Abbreviation: cSDS, conditional standard deviation score. The bold text was used to identify the cSDS column as being the most important.

## 7 DISCUSSION

In this paper, we have outlined a method for obtaining a single correlation matrix by combining a set of matrices from different studies. The innovation of this approach lies in its ability to combine a set of matrices that are heterogeneous in terms of the age range over which they are measured, while still maintaining the symmetry and completeness of the final correlation matrix. We achieve this via a 2‐stage process—first, we construct a raw, incomplete matrix by conducting a set of entrywise univariate meta‐analyses of the correlation matrices, and then we smooth the resulting matrix in order to produce a complete and valid correlation matrix. This represents a quick and relatively simple solution to a challenging problem. We appreciate that a multivariate solution may offer a number of advantages over this 2‐stage approach, but we also believe that adopting such an approach would come at the cost of sacrificing the simplicity and speed of our approach.

The motivation for this methodology was to provide a correlation matrix that explained the growth pattern of young children in resource‐poor settings. These studies are typically from countries with a low Human Development Index, and in most cases, the populations of these studies exhibit growth that is substantially lower than the global average. Existing charts and models for childhood growth have been designed to compare children with the global average, and these may not be appropriate for the children in our studies. We have thus produced a pair of correlation matrices for HAZ and WAZ within our study populations. This facilitates the calculation of centile‐crossing velocity scores such as the cSDS. These matrices also provide a very useful tool for health professionals who wish to monitor the growth and development of children in these countries. This work considers height and weight separately and constructs separate height × age and weight × age correlation matrices. However, it may be of interest in future to combine these into a single height × weight × age matrix, which could describe all possible growth relationships.

## Supporting information

Supporting info item

Supporting info item

Supporting info item

Supporting info item

## APPENDIX A FUNCTIONAL DATA ANALYSIS FOR LONGITUDINAL DATA

Consider the longitudinal data of the form {(*t*_*i**j*_,*Z*_*i**j*_),1≤*j*≤*m*_*i*_,1≤*i*≤*n*}, where *Z*_*i**j*_ (can be either HAZ or WAZ) is measured at time *t*_*i**j*_, *m*_*i*_ is the number of measurements for subject *i*, and *n* is the number of subjects. Then, the functional data model^31^, ^41^ for *Z*_*i**j*_ is

(A1) $$Z_{ij}=\mu (t_{ij})+b_{i}(t_{ij})+\varepsilon_{ij}.$$

Here, *μ*(*t*) is a fixed smooth mean function of *t*; *b*_*i*_(*t*) is a random function modelled by a zero‐mean Gaussian process with covariance operator $C(s,t)=\text{Cov}\{b_{i}(s),b_{i}(t)\}$; and *ε*_*i**j*_ is white noise with variance $\sigma_{\varepsilon }^{2}$. It is assumed that *b*_*i*_(*t*) and *ε*_*i**j*_ are independent within and across subjects.

Under model (A1), for a new subject with observations *Z*(*t*), the correlation between *Z*(*t*_1_) and *Z*(*t*_2_) at 2 time points *t*_1_ and *t*_2_ is

(A2) $$r(t_{1},t_{2})=\text{Cor}\{Z(t_{1}),Z(t_{2})\}=\frac{C(t_{1},t_{2})+1_{\left\{t_{1}=t_{2}\right\}}}{\sqrt{C(t_{1},t_{1})+\sigma_{\varepsilon }^{2}}\sqrt{C(t_{2},t_{2})+\sigma_{\varepsilon }^{2}}},$$

where 1_{·}_ is an indicator function that is equal to 1 if the statement inside the bracket is true and 0 otherwise. Thus, to estimate *r*(*s*,*t*), we just plug in estimates of C(s,t) and $\sigma_{\varepsilon }^{2}$ into (A2).

An estimate of *μ*(*t*) can be obtained by univariate smoothing of *Z*_*i**j*_, ignoring within‐subject correlations; denote the estimate by $\hat{\mu }(t)$. Then, empirical estimates of the covariance function can be constructed by $r_{ij_{1}j_{2}}=(Z_{ij_{1}}-\hat{\mu }(t_{ij_{1}}))(Z_{ij_{2}}-\hat{\mu }(t_{ij_{2}})),1\leq j_{1}\leq j_{2}\leq m_{i},1\leq i\leq n$. A bivariate smoothing of $r_{ij_{1}j_{2}}$ provides a smooth estimate of the covariance function and also an estimate of $\sigma_{\varepsilon }^{2}$.

## APPENDIX B BIVARIATE P‐SPLINE SMOOTHER

To obtain smooth estimates of correlation functions on the off‐diagonals and also to fill in unobserved correlations, we conduct a bivariate smoothing on the existing correlations. Let $({\tilde{h}}_{ij},a_{i},a_{j},\delta_{ij}),1\leq i\leq r,1\leq j\leq r$ be the estimated correlations from stage 1, where ${\tilde{h}}_{ij}$ is the estimated correlation at ages *a*_*i*_ and *a*_*j*_ and *δ*_*i**j*_ is 1 if ${\tilde{h}}_{ij}$ exists and 0 otherwise.

We first conduct a Fisher transformation of these correlations, $g_{ij}=\frac{1}{2}\ln\left(\frac{1-{\tilde{h}}_{ij}}{1+{\tilde{h}}_{ij}}\right)$. Then, we conduct a bivariate smoothing of *g*_*i**j*_ under the working model *g*_*i**j*_=*g*(*a*_*i*_,*a*_*j*_)+*ε*_*i**j*_, where *g*(*a*_*i*_,*a*_*j*_) is a bivariate smooth function and *ε*_*i**j*_ is independent Gaussian random variables. We use the spline smoother outlined in Marx and Eilers,^38^ with a constraint added to ensure the symmetry of our eventual correlation function. We obtain an estimated function $Ĝ(a_{i},a_{j})$ and apply the inverse Fisher transform to $Ĝ(a_{i},a_{j})$ to obtain our correlation estimate ${\hat{\rho }}_{ij}$.

The bivariate *P*‐spline smoother approximates *g*(*a*_*i*_,*a*_*j*_) by a set of tensor‐product splines *G*(*a*_*i*_,*a*_*j*_)= ∑_1≤*κ*≤*c*,1≤*ℓ*≤*c*_ *θ*_*κ**ℓ*_ *B*_*κ*_(*a*_*i*_)*B*_*ℓ*_(*a*_*j*_), where **Θ**=(*θ*_*κ**ℓ*_)_1≤*κ*≤*c*,1≤*ℓ*≤*c*_ is a coefficient matrix; {*B*_1_(·),…,*B*_*c*_(·)} is the collection of univariate B‐spline basis functions; and *c* is the number of basis functions. Twenty cubic B‐splines is used on each dimension so that the potentially complex nature of correlations can be captured by a large number of basis functions (400 in total). The following constraint on **Θ** is also enforced:

$$\theta_{\kappa \ell }=\theta_{\ell \kappa },1\leq \kappa ,\ell \leq c.$$

With this constraint, *G*(*a*_*i*_,*a*_*j*_) is always symmetric, a desired property for estimates of correlation functions.

With a large number of basis functions, estimating **Θ** by least squares tends to overfit. Thus, following Marx and Eilers,^38^ we estimate **Θ** by minimising the penalised least squares:

$$\frac{1}{r^{2}}\sum_{i=1}^{r}\sum_{j=1}^{r}\delta_{ij}{\left\{g_{ij}-\sum_{1\leq \kappa \leq c,1\leq \ell \leq c}\theta_{\kappa \ell }B_{\kappa }(a_{i})B_{\ell }(a_{j})\right\}}^{2}+\lambda P(\Theta ),$$

where *P*(**Θ**) is the penalty used in Marx and Eilers^38^ and is essentially equivalent to the penalty $\iint_{a,b}{\left\{\frac{\partial^{2}G(a,b)}{\partial a^{2}}\right\}}^{2}dadb=\iint_{a,b}{\left\{\frac{\partial^{2}G(a,b)}{\partial b^{2}}\right\}}^{2}dadb$ and *λ* is a tuning parameter that balances model fit and smoothness of estimate and can be selected by cross‐validation methods. Then, we estimate *g*(*a*_*i*_,*a*_*j*_) by $Ĝ(a_{i},a_{j})=\sum_{1\leq \kappa \leq c,1\leq \ell \leq c}{\hat{\theta }}_{\kappa \ell }B_{\kappa }(a_{i})B_{\ell }(a_{j})$ and apply the inverse Fisher transform to $Ĝ(a_{i},a_{j})$ to obtain ${\hat{\rho }}_{ij}=\frac{\exp\{2Ĝ(a_{i},a_{j})\}-1}{\exp\{2Ĝ(a_{i},a_{j})\}+1}$.

We note that this penalised least squares formula incorporates a constant variance term *τ* ^2^, thus implying that the variance across the studies is independent of time. The model could be extended to incorporate a term $\tau_{i,j}^{2}$, which allows for different variances across different combinations of time points *i* and *j*. This approach has not been considered here but could merit further exploration in the future.

To ensure the integrity of the correlation matrix, we remove the diagonal elements (which are 1s) prior to the smoothing and then normalise the smoothed correlations to ensure that the final matrix has 1s on the diagonal. Specifically, let $\hat{\Sigma }=({\hat{\rho }}_{ij})$ be the estimated correlation matrix after smoothing; then, the final matrix is $\tilde{\Sigma }=({\tilde{\rho }}_{ij})$, where ${\tilde{\rho }}_{ij}={\hat{\rho }}_{ij}/\sqrt{{\hat{\rho }}_{ii}{\hat{\rho }}_{jj}}$.
