# Supplementary figures and images for: Using data from multiple studies to develop a child growth correlation matrix
Source: Stat Med. 2018 Apr 26;38(19):3540–54. doi: 10.1002/sim.7696 (PMC6767589; doi:10.1002/sim.7696)

WAZ: Upper Bound

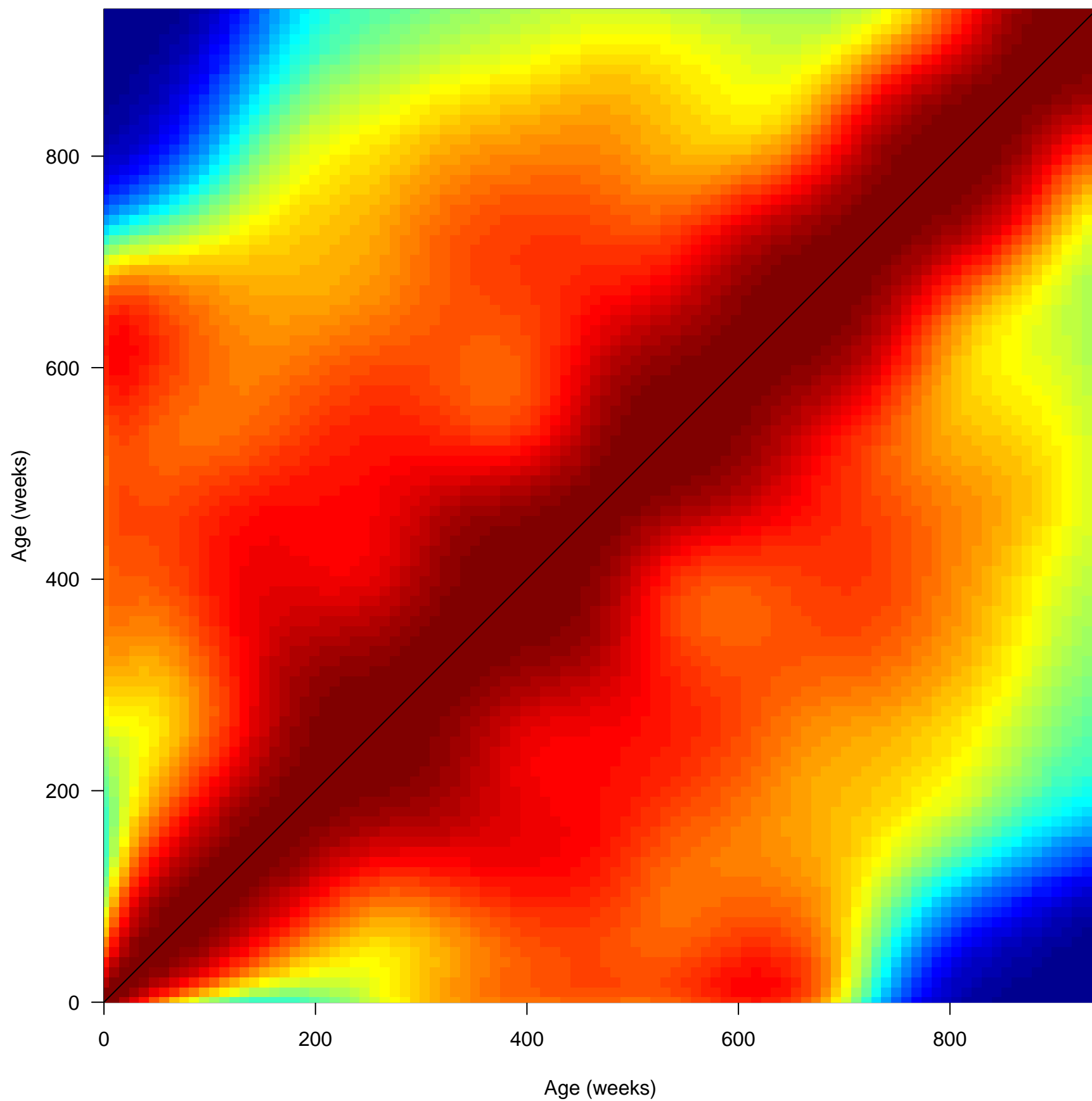

Supplement: Supplementary file 1 — Supporting info item [file SIM-38-3540-s001.pdf]

# WAZ: Incomplete

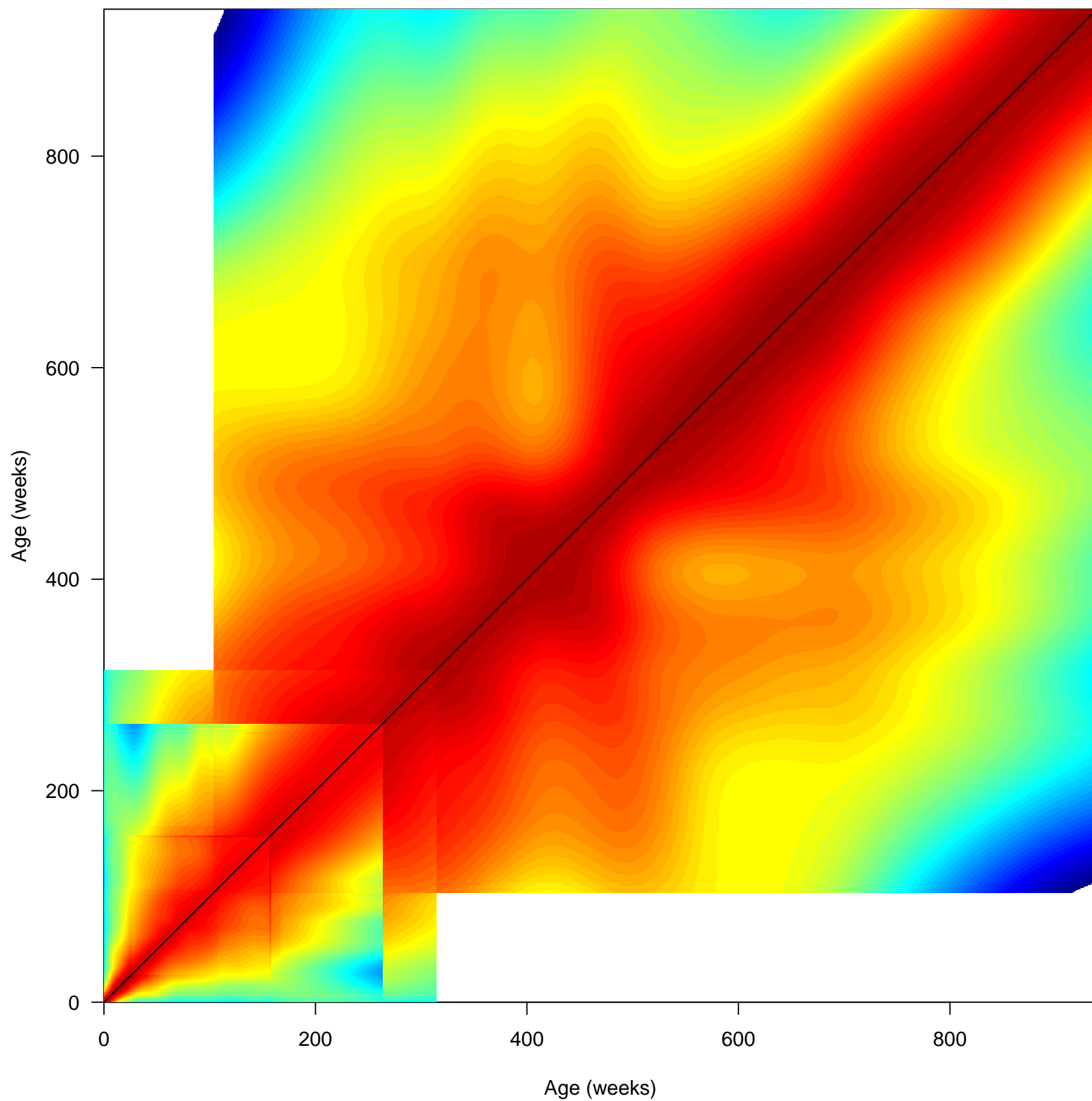

Supplement: Supplementary file 2 — Supporting info item [file SIM-38-3540-s002.pdf]

**WAZ: Lower Bound**

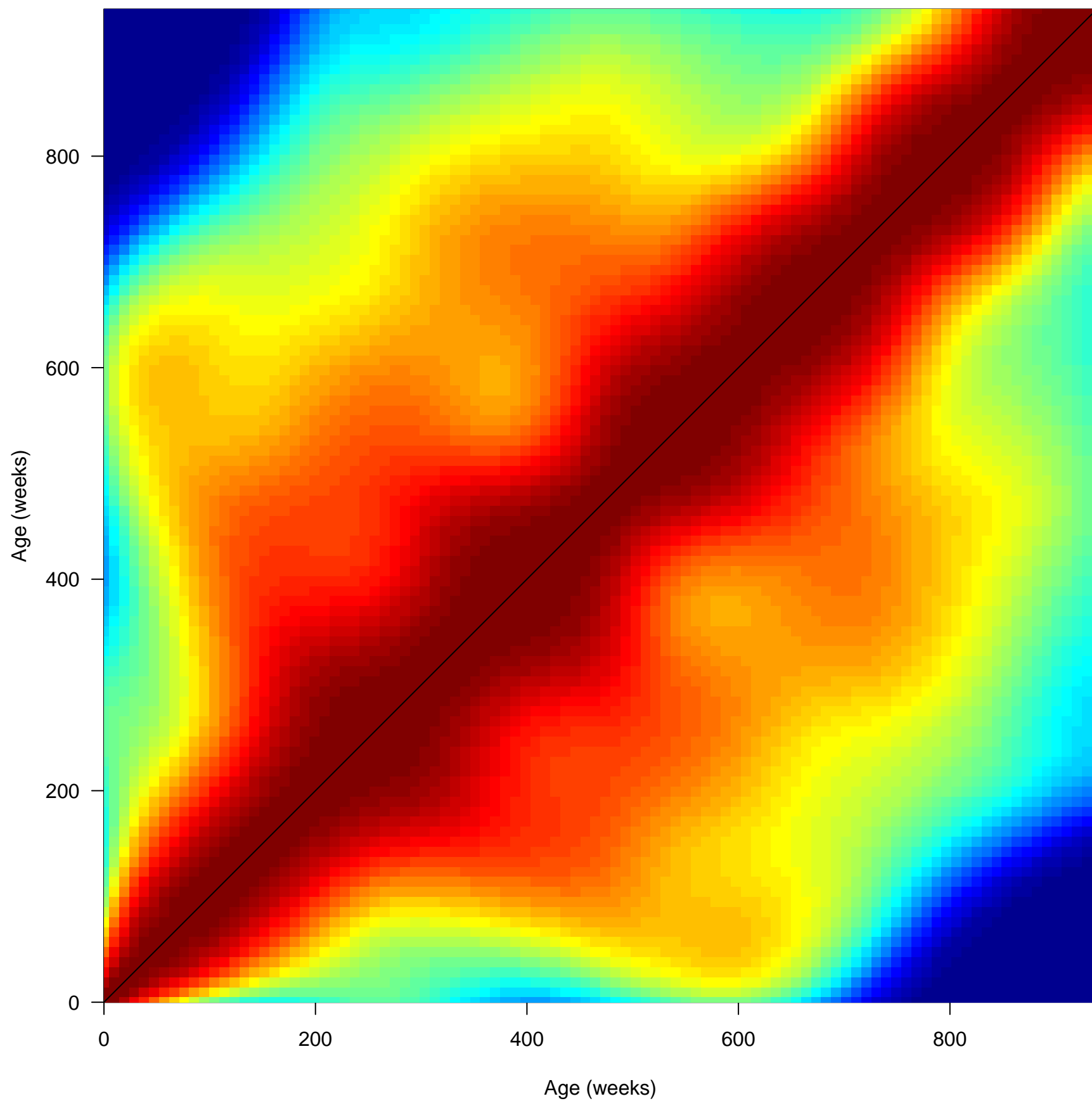

Supplement: Supplementary file 3 — Supporting info item [file SIM-38-3540-s003.pdf]

WAZ: Complete After Smoothing

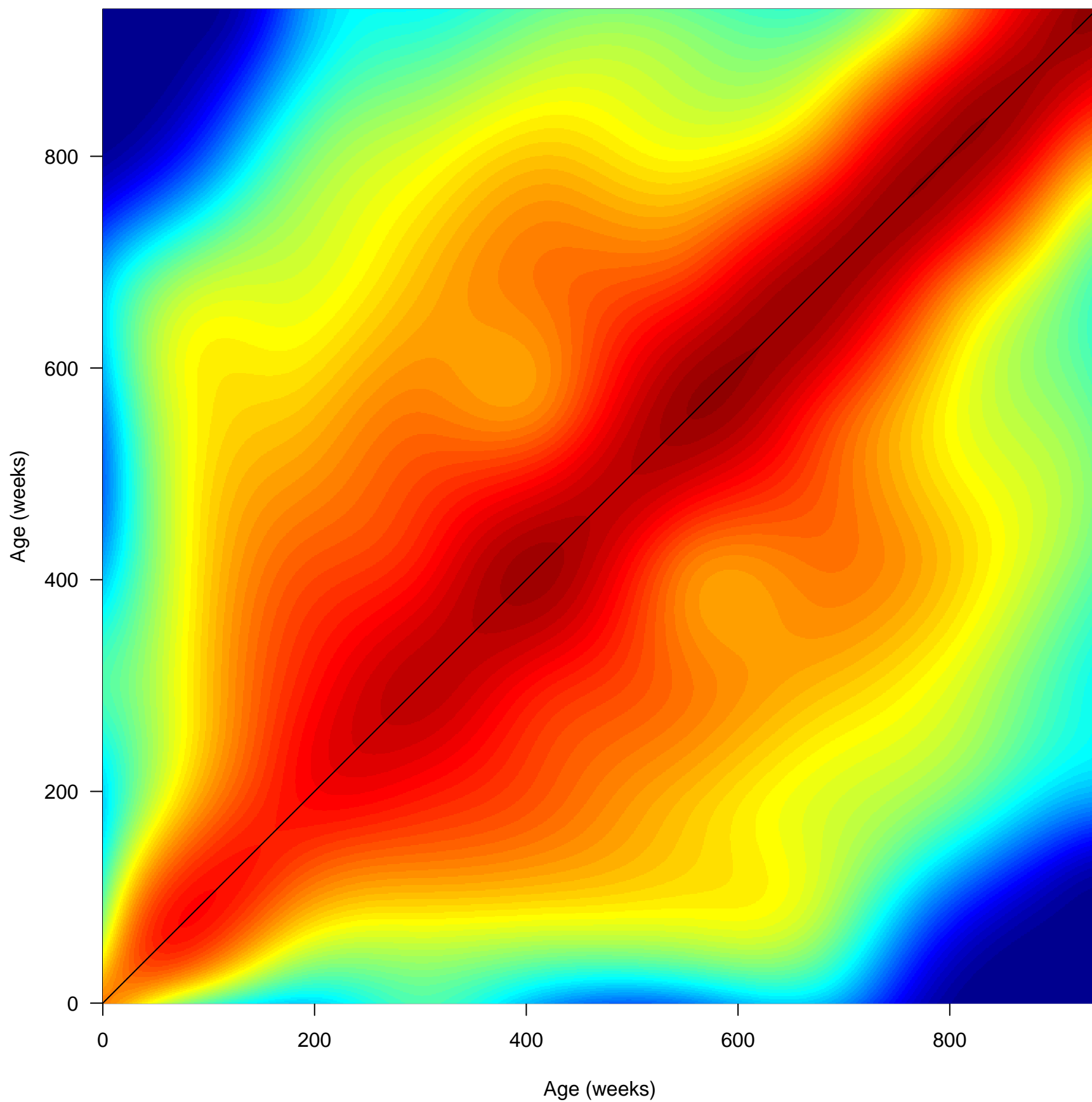

Supplement: Supplementary file 4 — Supporting info item [file SIM-38-3540-s004.pdf]
